# Flash Memory for Synaptic Plasticity in Neuromorphic Computing: A Review

**DOI:** 10.3390/biomimetics10020121

**Published:** 2025-02-18

**Authors:** Jisung Im, Sangyeon Pak, Sung-Yun Woo, Wonjun Shin, Sung-Tae Lee

**Affiliations:** 1 School of Electronic and Electrical Engineering, Kyungpook National University, Daegu 41566, Republic of Korea; wltjd7055@naver.com; 2 School of Electronic and Electrical Engineering, Hongik University, Seoul 04066, Republic of Korea; spak@hongik.ac.kr; 3 Department of Semiconductor Convergence Engineering, Sungkyunkwan University, Suwon 16419, Republic of Korea

**Keywords:** neuromorphic, synaptic device, in-memory computing, NOR flash memory, AND flash memory, NAND flash memory, flash memory

## Abstract

The rapid expansion of data has made global access easier, but it also demands increasing amounts of energy for data storage and processing. In response, neuromorphic electronics, inspired by the functionality of biological neurons and synapses, have emerged as a growing area of research. These devices enable in-memory computing, helping to overcome the “von Neumann bottleneck”, a limitation caused by the separation of memory and processing units in traditional von Neumann architecture. By leveraging multi-bit non-volatility, biologically inspired features, and Ohm’s law, synaptic devices show great potential for reducing energy consumption in multiplication and accumulation operations. Within the various non-volatile memory technologies available, flash memory stands out as a highly competitive option for storing large volumes of data. This review highlights recent advancements in neuromorphic computing that utilize NOR, AND, and NAND flash memory. This review also delves into the array architecture, operational methods, and electrical properties of NOR, AND, and NAND flash memory, emphasizing its application in different neural network designs. By providing a detailed overview of flash memory-based neuromorphic computing, this review offers valuable insights into optimizing its use across diverse applications.

## 1. Introduction

Artificial intelligence (AI) has recently achieved remarkable advancements across various cognitive tasks, including natural image classification, speech recognition, and language processing [1,2,3,4,5,6,7,8]. However, achieving such a high performance with deep neural networks (DNNs) demands significantly larger network sizes and an increased number of parameters. This necessitates the use of high-performance graphics processing units (GPUs), substantial memory, and extensive computational resources [9,10,11]. Additionally, the von Neumann bottleneck—caused by frequent data transfers between memory and processors—results in considerable time and energy inefficiencies, particularly during multiplication and accumulation (MAC) operations.

Neuromorphic systems utilizing synaptic device arrays have been widely investigated as a means to address the memory bandwidth limitation by enabling in-memory computation [12,13,14]. These arrays perform VMM efficiently by exploiting the productof the conductance of memory cells and input voltages, where the resulting currents are aggregated. This parallel summation of currents across source lines within a single time-step enhances computational efficiency compared to traditional architectures.

Research efforts have predominantly focused on resistive random-access memory (RRAM) [15,16,17,18,19] and phase-change memory (PCM) [20,21,22,23,24,25,26] as neuromorphic electronics. On the other hand, RRAM faces challenges such as the integration of selectors for massive RRAM array, device variability, reliability concerns, and stochastic programming. The small on/off resistance ratio of RRAM and voltage drops in metal wires further compromise VMM accuracy. Similarly, PCM suffers from resistance drift in its amorphous chalcogenide material, posing significant reliability issues, particularly in multi-level cell (MLC) operations [27,28]. Recently, state-of-the-art technologies provided the benefits of ferroelectric memories, which have the potential to be used in neuromorphic computing [29,30].

To address the limitations mentioned above, mature silicon-based devices like static random-access memory (SRAM) [31,32,33,34,35,36,37,38,39,40] have been investigated as alternative synaptic devices. However, these technologies are constrained by low integration density due to their large cell size. Flash memory, in contrast, offers a high integration density and substantial storage capacity per chip, making it an attractive option for handling the extensive parameters required by state-of-the-art DNNs [41,42,43,44,45,46,47,48,49]. Moreover, flash memory has been demonstrated to be both commercially competitive and technologically mature [50,51]. Therefore, flash memory has emerged as a promising candidate for neuromorphic computing due to its scalability and technological maturity. In addition, flash memory can be used in photonic programming and in-sensor computing [52,53].

This review provides a detailed exploration of recent advancements in flash memory-based neuromorphic computing. It examines neuromorphic architectures for NOR flash memory [54,55,56,57,58,59,60], AND flash memory [61,62,63,64,65,66], and NAND flash memory [67,68,69,70,71,72,73,74] in off-chip and on-chip learning, with a focus on array architectures, operational methodologies, and performance characteristics. By highlighting the characteristics of NOR flash memory, AND flash memory, and NAND flash memory, this article lays the groundwork for understanding flash memory’s role in neuromorphic computing and offers valuable insights into its potential applications in neural network designs.

## 2. Neuromorphic Computing Based on NOR Flash Memory

**Figure 1:**
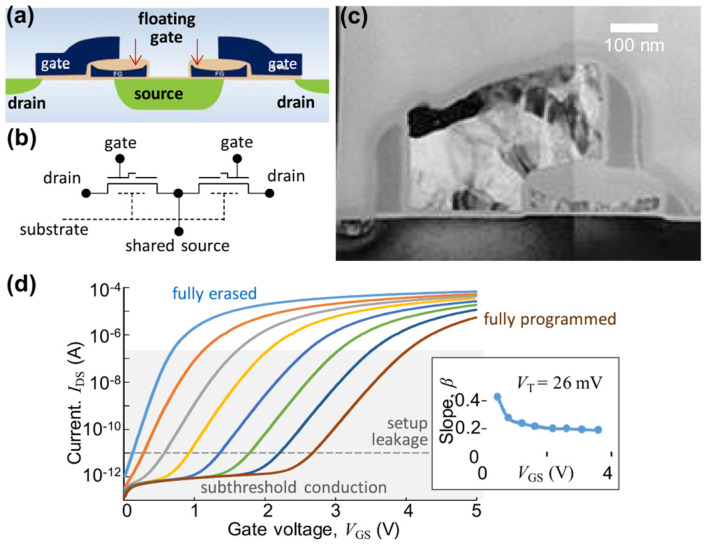
ESF1 NOR flash memory cells. (**a**) Schematic cross-section of the two-cell “supercell” structure and (**b**) its corresponding equivalent circuit. (**c**) TEM cross-sectional image of an individual memory cell fabricated using a 180 nm process. (**d**) The drain current (*I*_DS_) of the memory cell is plotted as a function of gate voltage (*V*_GS_) at *V*_DS_ = 1 V for various memory states. Lines of different colors have different threshold voltages, which result from the program and erase pulses applied to the NAND cell. The gray-shaded area indicates the subthreshold conduction region, where currents below *I*_DS_ = 10 pA (dashed line) are predominantly affected by leakage currents from the experimental setup used for measurements. Inset: Slope values extracted from the semilogarithmic plot at *I*_DS_ = 10 nA, shown as a function of the memory state, characterized by the corresponding gate voltage. Reprinted/adapted with permission from Ref. [54]. Copyright © 2017, IEEE.

Bayat et al. introduced a high-performance mixed-signal neurocomputing approach utilizing nanoscale floating-gate memory cell arrays [54]. The network architecture presented in this study incorporates an energy-efficient gate-coupling mechanism [75,76,77,78], which operates effectively in the subthreshold mode (Figure 1). In this mode, the drain current (*I*_DS_) of the memory cell exhibits an almost exponential relationship with the gate voltage (*V*_GS_), as depicted in Figure 1d.

Notably, the inset in Figure 1d highlights that the slope of this relationship remains relatively stable across a wide range of memory states for ESF1 cells, enabling reliable gate-coupled circuit functionality.

**Figure 2:**
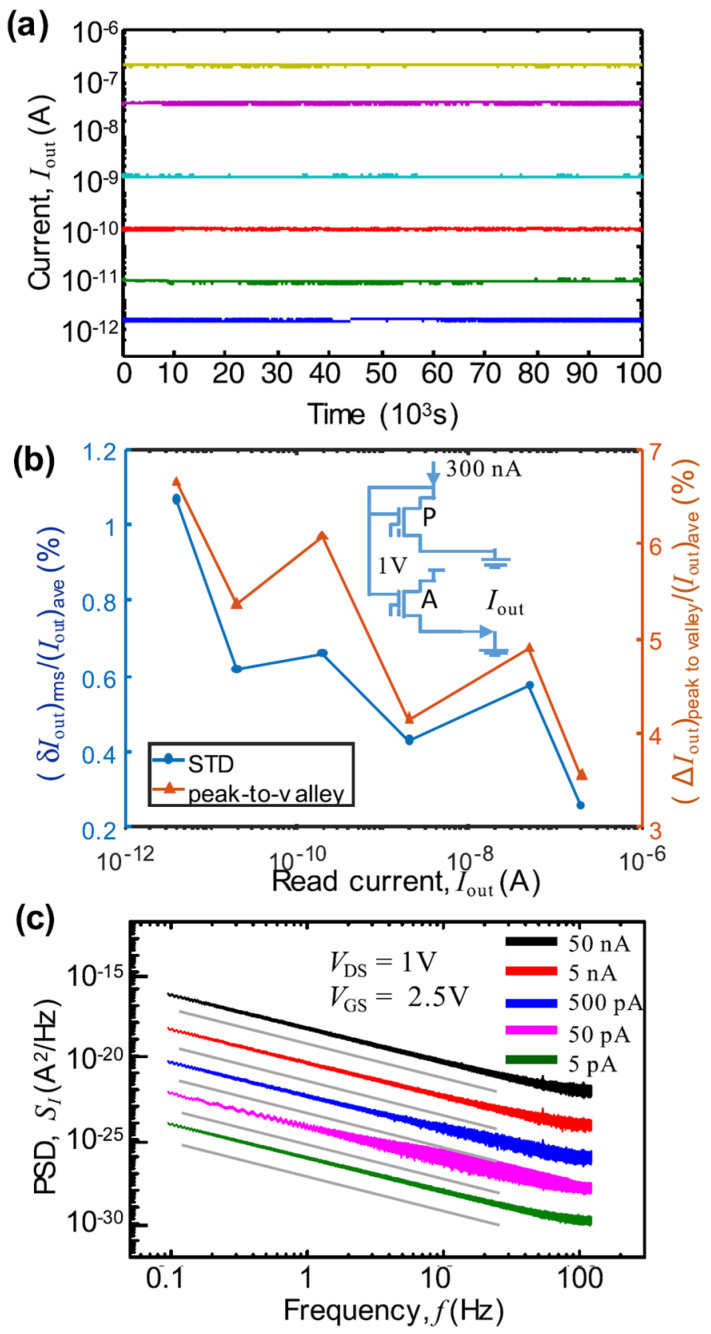
(**a**) Results of analog retention measurements for various memory states conducted in the gate-coupled array configuration. Each state is represented by 1000 data points, where each point is the average of 65 samples collected over a 130 ms interval. Lines of different colors represent the retiontion characteristics with different current level. (**b**) Relative root mean square (rms) variation and the full peak-to-valley current swing during the same measurement period. The inset shows the equivalent circuit of the gate-coupling configuration used. (**c**) Spectral density of the cell current noise measured at room temperature. The gray lines serve as visual guides, corresponding to *S*_I_ ∝ 1/*f*^1.6^. Reprinted/adapted with permission from Ref. [54]. Copyright © 2017, IEEE.

To maintain relative current fluctuations within 1%, the subthreshold operation achieves a dynamic range spanning approximately five orders of magnitude, from ~10 pA to ~300 nA, corresponding to a gate voltage variation of ~1.5 V (Figure 2b). The ESF1 flash technology ensures a 10-year retention in digital mode at temperatures as high as 125 °C [79]. Experimental results further indicate that these cells exhibit analog-level retention for at least several days, with minimal output-current fluctuations (Figure 2a). Additional testing of analog-level retention, conducted on 55 nm ESF3 NOR cells fabricated with similar technology, demonstrated negligible drift in memory states for nearly one day, even at an elevated temperature of 85 °C.

Other characteristics of the ESF1 cell arrays, such as modifications, switching dynamics, statistical behavior, and fast weight tuning with an accuracy of ~0.3%, have been detailed in previous studies [80].

**Figure 3:**
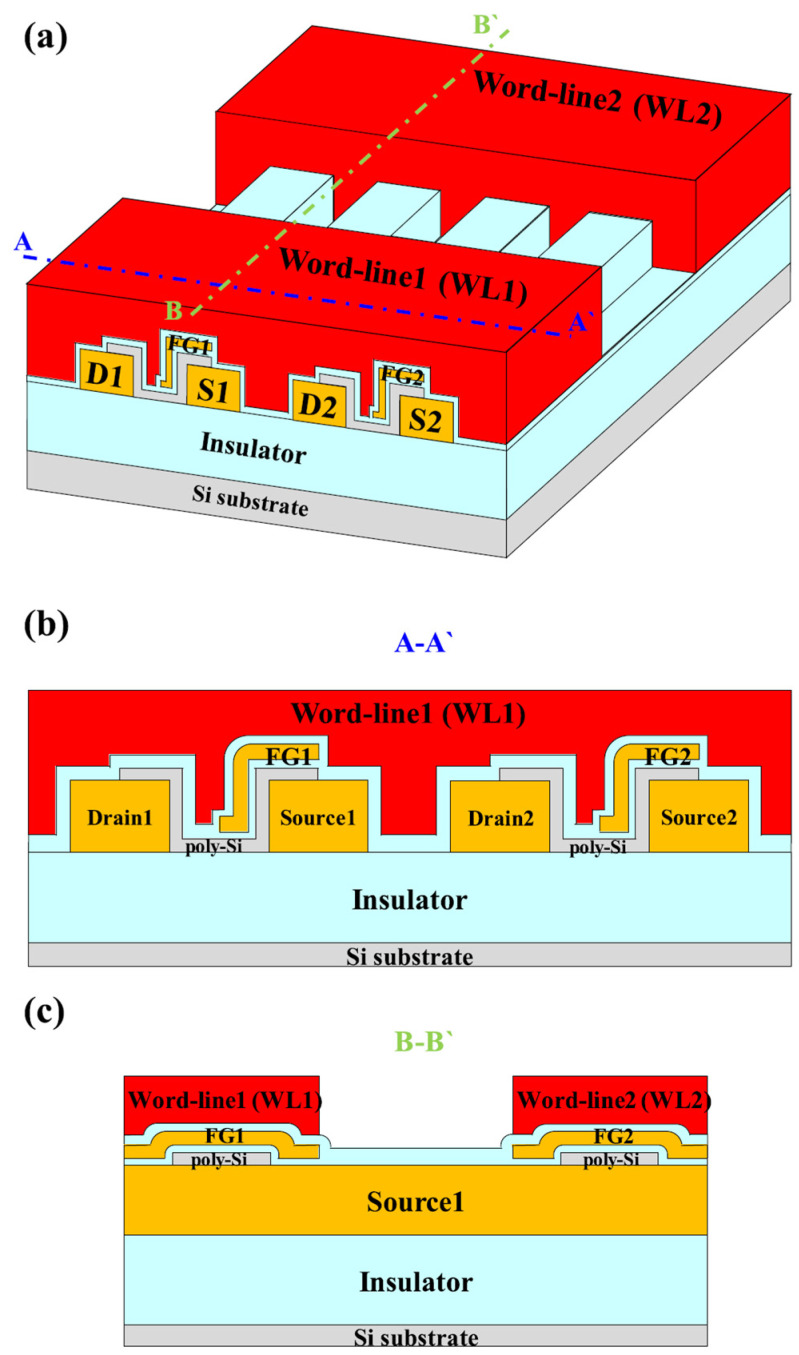
(**a**) Top-down view of a synapse cell array, with cross-sectional views shown in (**b**) along the WL direction and (**c**) along the BL direction. Reprinted/adapted with permission from Ref. [55]. Copyright © 2018, IEEE.

Kim et al. demonstrated unsupervised learning utilizing spike-timing-dependent plasticity in a TFT-type NOR flash memory array [55]. Figure 3a provides a top-down view of this memory array, while cross-sectional views along A–A’ and B–B’ are illustrated in Figure 3b and Figure 3c, respectively. As shown in Figure 3a, the word-lines (WLs) and bit-lines (BLs) form a crossbar structure, allowing efficient scaling of large memory arrays. In Figure 3b, each device’s drain and source are connected through a poly-Si channel, partially covered by an *n*^+^ poly-Si floating gate (FG) that is separated by an interpoly dielectric layer. A single WL simultaneously controls multiple devices. The FG, positioned between the WL and the source, enables programming and erasing operations when a voltage is applied between these electrodes. This partial FG coverage ensures that the threshold voltage (Vt) remains above zero in the fully erased state, effectively preventing leakage currents and lowering standby power consumption by mitigating excessive synaptic potentiation during unsupervised learning.

In Figure 3c, the structural arrangement of the device array along BL direction is illustrated. The adjacent floating gates (FGs) are electrically isolated, ensuring that each device can operate independently for memory functions. The drain and source lines are positioned in parallel along the BL direction and are shared among *n* memory cells under *n* WLs, allowing the cumulative summation of currents from *n* NOR flash memory cells. This architecture enables each flash cell to transfer its stored data to the BL as a combined current, resembling biological synapses, where individual synapses contribute their weight information to the overall signal transmitted to the subsequent neuron.

**Figure 4:**
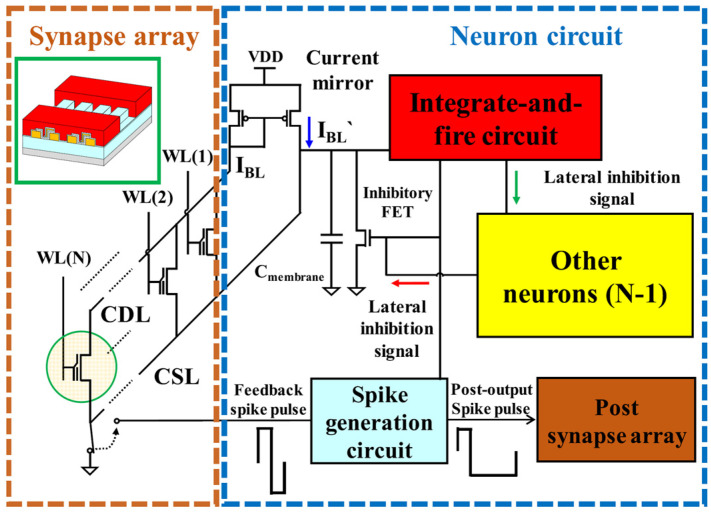
Schematic circuit diagram of a STDP neuromorphic network incorporating a synapse cell array and neuron circuitry. Reprinted/adapted with permission from Ref. [55]. Copyright © 2018, IEEE.

Figure 4 depicts the circuit design of a neural network system utilizing a TFT-type NOR flash memory array. Input representations transferred from the PRE neurons are imposed on the WLs of the flash cells. These WL signals are transformed into currents that correspond to the synaptic weights stored within the array and are subsequently aggregated along the common drain line (CDL). The total current accumulated in the CDL is then relayed to the POST neuron circuitry outside the array through a current mirror circuit.

Within the POST neuron, the current charges a membrane capacitor, triggering neuron activation when the membrane potential surpasses a predefined threshold. To ensure selective activation, each POST neuron incorporates an FET-type inhibitory synapse that suppresses the firing of other neurons. When the POST neuron fires, it activates a switch that connects the common source line (CSL) to the ground, enabling interaction with the spike generation circuit. Additionally, the firing signal is transmitted to the spike generation circuit, which generates a feedback spike pulse to the CSL of its own array and an output spike pulse to the WLs of the synapse array in the subsequent neural network layer. This design facilitates synaptic weight updates on a neuron-by-neuron basis without necessitating extra circuitry or sequential updating processes.

**Figure 5:**
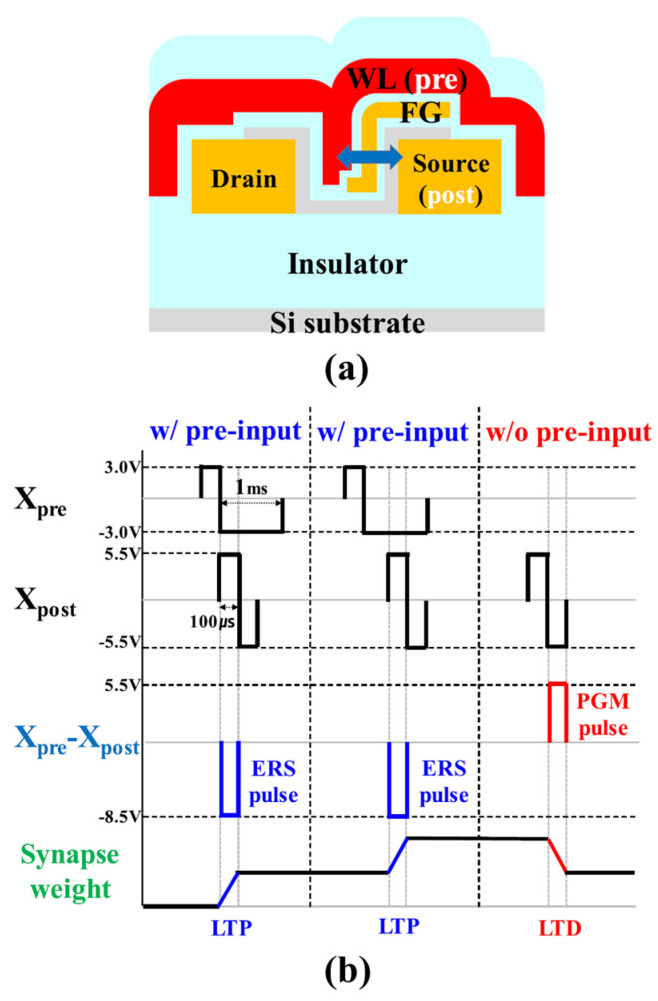
(**a**) Schematic of PRE (input) and POST (feedback) electrodes responsible for updating synaptic weights, and (**b**) the pulse scheme applied to the synapse cellarray, enabling LTP and LTD through the erase (ERS) and program (PGM) operations of the memory cell. Reprinted/adapted with permission from Ref. [55]. Copyright © 2018, IEEE.

For spike-timing-dependent plasticity (STDP) operations, flash cells can be selectively potentiated or depressed utilizing the pulse scheme illustrated in Figure 5. The underlying mechanism governing long-term potentiation (LTP) and long-term depression (LTD) in synaptic cells functions as follows: when a neuron fires, the synapse cells involved in this firing undergo the LTP process through the overlap of incoming signals and feedback signals from the neuron. Conversely, synapse cells that do not receive an input signal are subjected to the LTD process via the feedback signal alone. This type of pulse scheme has been previously explored in [81].

As illustrated in Figure 5a, input voltages from PRE neurons are imposed on the WLs, while feedback signals generated by the spike generation circuit are delivered to the CSL to adjust synaptic weights. The modification of charge within the FG is determined by the voltage states of the source connected to the CSL and the WL. During LTP, when an input pulse initiates neuron firing, the leading edge of the feedback pulse overlaps with the trailing edge of the input pulse, as depicted in Figure 5b. To simulate the LTP mechanism, a −8.5 V pulse (*X*_pre_−*X*_post_ in Figure 5b) is imposed on the WL for 100 μs, triggering an erase operation in the FG.

Conversely, when no input signal is received from the PRE neurons, only the feedback pulse is imposed on the memory cell’s source. In this case, a pulse of 5.5 V with a 100 μs duration is imposed on the WL, facilitating the programming of the FG by storing electrons, thereby replicating the LTD behavior of the synapse.

## 3. Neuromorphic Computing Based on AND Flash Memory

Kim et al. proposed a vertical AND-type flash synaptic cell stack for high-density and reliable binary neural networks [61].

**Figure 6:**
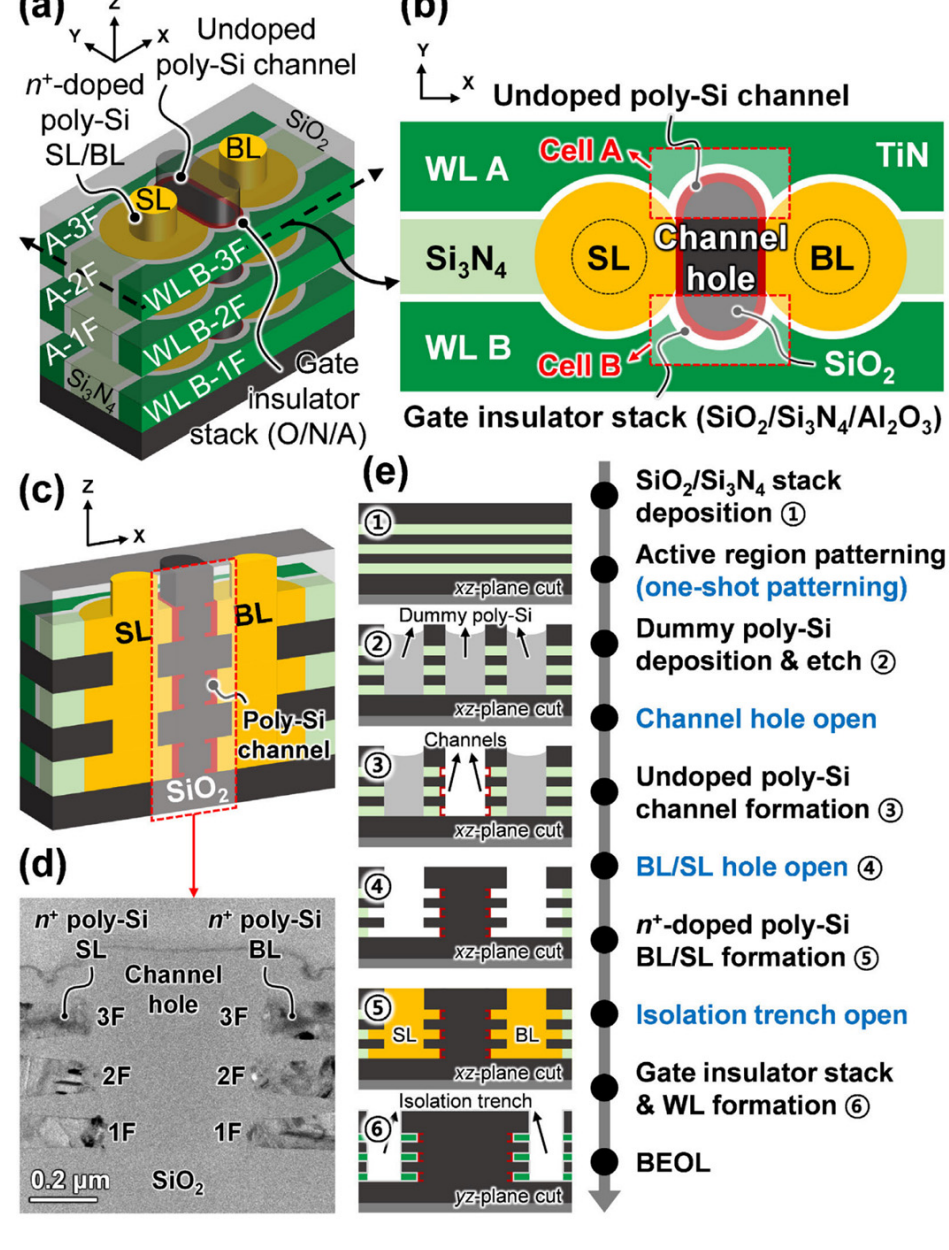
(**a**) Schematic representation of a V-AND flash memory cell stack, with cross-sectional views shown in (**b**) the xy plane and (**c**) the xz plane. (**d**) TEM cross-sectional image of the fabricated V-AND cell stack. (**e**) Key fabrication steps for the V-AND cell stack, including one-shot patterning. Reprinted/adapted with permission from Ref. [61]. Copyright © 2024, IEEE.

Figure 6a represents a schematic view of a V-AND flash memory cell stack. Within the vertical plug-shaped channel hole, an undoped poly-Si channel is created, surrounded by a gate insulator stack (SiO₂/Si₃N₄/Al₂O₃) and TiN word-lines (WLs). Bit-lines (BLs) and source-lines (SLs), composed of *n*^+^-doped poly-Si, are positioned on both sides of the channel hole along the *x*-axis. These vertical plug-shaped BLs and SLs interconnect the flash memory cells aligned along the vertical axis, forming a vertically oriented AND array structure. Cross-sectional views in the xy and xz planes are shown in Figure 6b and Figure 6c, respectively. In each layer, pairs of cells (A and B) share a source/drain and face each other along the channel hole’s long axis. Each cell is independently controlled by separate WLs (WLs A and B), separated by a Si₃N₄ region. This design significantly enhances the cell density of the V-AND flash memory, while the vertically separated channels minimize interference between cells along the vertical axis, as illustrated in Figure 6c. A transmission electron microscopy (TEM) image of the fabricated V-AND flash cells, achieving a density of ~3.67 F^2^ per cell, is shown in Figure 6d. Figure 6e outlines the key fabrication steps for the proposed V-AND flash memory cell stack. First, multiple layers of SiO₂/Si₃N₄ are sequentially deposited. A single-patterning process is then used to define channel holes, BL/SL holes, and isolation trenches, which separate V-AND cell stacks in the horizontal direction. Dummy poly-Si is deposited and planarized to fill the patterned holes. Channels, BL/SL, and WL electrodes are formed sequentially, with selective opening and removal of the dummy poly-Si at each step. Si₃N₄ layers exposed on the hole surfaces are selectively etched, creating channels and electrodes in these regions. Channels for each floor are separated through anisotropic etching, and the process concludes with the back-end-of-line (BEOL) fabrication. Although Figure 6 shows a three-floor V-AND cell stack as an example, additional layers can be stacked to further enhance cell density. Utilizing equipment and process recipes from V-NAND mass production allows for the fabrication of cells with many more layers than demonstrated in this study.

**Figure 7:**
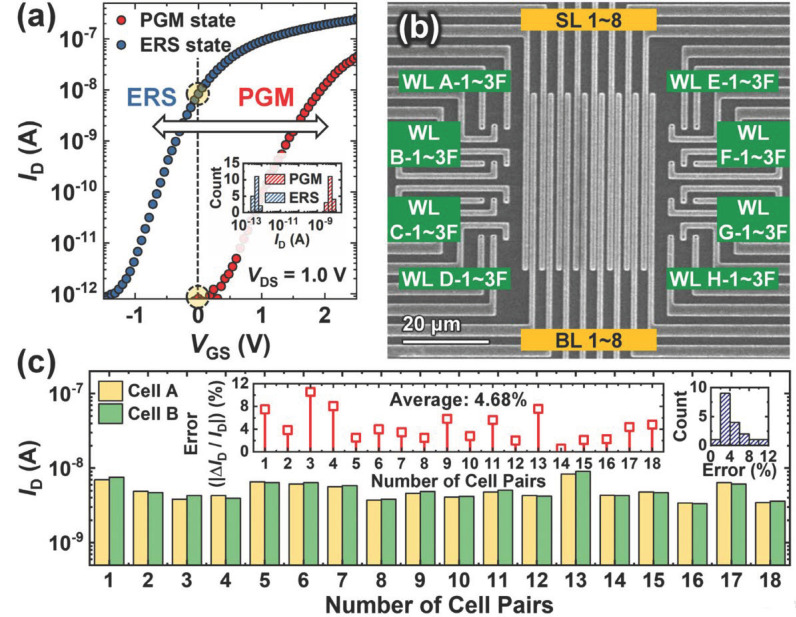
(**a**) Transfer curves (*I*_D_-*V*_GS_) of the fabricated V-AND flash cell in PGM and ERS states. The inset shows the *I*_D_ distribution for the ERS and PGM states (*t*_read_ = 100 μs, *t*_write_ = 1 ms). (**b**) Top-view SEM image of the 8 × 8 × 3 V-AND flash array. (**c**) Measured *I*_D_ values for 18 randomly selected cell pairs (cells A and B) sharing a single channel hole and source/drain at *V*_GS_ = 0 V, showing similar *I*_D_ values between paired cells. Insets display the ratio of the *I*_D_ difference (Δ*I*_D_) between cells A and B and its distribution. Reprinted/adapted with permission from Ref. [61]. Copyright © 2024, IEEE.

To assess the synaptic characteristics of the fabricated V-AND flash cells for binary neural networks (BNNs), the drain current (*I*_D_) of the cells is measured. Figure 7a shows the transfer curves (*I*_D_-*V*_GS_) of the fabricated cell in both the program (PGM) and erase (ERS) states. The cells demonstrate a high on/off current ratio exceeding 10^5^, with an off current in the sub-pA range under *V*_DS_ = 1.0 V. These PGM and ERS states were achieved using low-amplitude pulses (8.0 V), which were made possible by the enhanced gate controllability provided by the V-AND flash cell’s gate structure surrounding convex channel. A dynamic range (*G*_max_/*G*_min_) of over 10^4^ was observed for the PGM/ERS states at *V*_GS_ = 0 V. This large dynamic range, along with a low off-current and wide memory window, is critical for the high performance of hardware-based BNNs. Additionally, the memory window can be further expanded by applying higher-amplitude PGM/ERS pulses. Figure 7b displays the top scanning electron microscopy (SEM) image of the fabricated V-AND flash array, which has a configuration of 8 × 8 × 3. Figure 7c shows the measured *I*_D_ values for 18 randomly selected pairs of cells (cells A and B) that share a single channel hole and source/drain. The results indicate no significant difference between the *I*_D_ values of cells A and B. The insets in Figure 7c illustrate the distribution and ratio of the *I*_D_ differences (Δ*I*_D_ = *I*_D,A_ − *I*_D,B_) between the two cells. The similarity in *I*_D_ values ensures reliable operation for hardware-based BNNs.

## 4. Neuromorphic Computing Based on NAND Flash Memory

**Figure 8:**
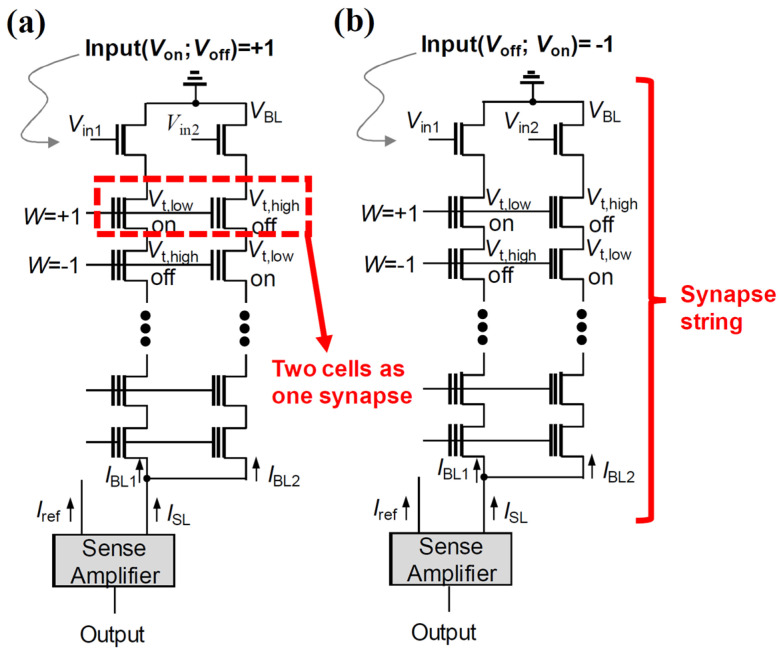
**A** 2T2S synaptic string structure, which consists of two input transistors and two NAND strings, along with a sense amplifier utilizing a fixed reference current (*I*_ref_) for a input value of (**a**) +1 and (**b**) −1. Reprinted/adapted with permission from Ref. [70]. Copyright © 2019, IEEE.

Lee et al. proposed a method to harness cell string structure as binary neuromorphic devices [67,70]. Figure 8 introduces a 2T2S (two transistors and two NAND cell strings) configuration based synaptic string structure, specifically designed for XNOR operations. In this design, each synapse string consists of two NAND cell strings, where each string is composed of serially connected NAND cells with two input transistors regulated by two distinct input voltages imposed on their gates. A synapse is formed by a pair of adjacent NAND cells from the two strings, representing a synaptic weight of +1 when the left NAND cell exhibits a low threshold voltage (*V*_th,low_) and the right NAND cell has a high threshold voltage (*V*_th,high_). Conversely, a synaptic weight of −1 is expressed when the threshold voltage states are reversed. Input values are defined using complementary input voltages: an input of +1 is achieved when *V*_in1_ and *V*_in2_ correspond to the turn-on voltage (*V*_on_) and turn-off voltage (*V*_off_), respectively. An input of −1 is defined by reversing this voltage pattern. In this approach, the string current (*I*_SL_), which corresponds to the XNOR output, is determined based on the combination of the states of two adjacent NAND flash cells and the complementary input voltages. The 2T2S structure functions entirely in the digital domain, eliminating the necessity for analog-to-digital converters (ADCs) or large operational amplifiers commonly required in analog vector–matrix multiplication (VMM) systems. Therefore, 2T2S design can significantly reduce power consumption compared to the analog VMM systems. In addition, as the number of stacks increases, the effective area of one synapse in V-NAND flash memory becomes smaller. The synapse density of V-NAND flash memory at a stack number of 128 is ~103 times higher than that of the RRAMs [67].

**Figure 9:**
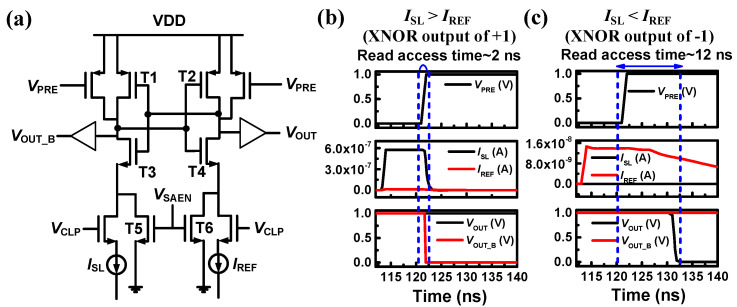
(**a**) The CSA compares the reference current (*I*_REF_) with the string current (*I*_SL_). transient waveforms of the CSA are shown for (**b**) a case where *I*_SL_ is larger than *I*_REF_ and (**c**) a case where *I*_SL_ is smaller than *I*_REF_. Reprinted/adapted with permission from Ref. [70]. Copyright © 2019, IEEE.

Figure 9a illustrates a current-latch-based CSA circuit tailored for BNNs. Given that the NAND cell’s *I*_on_ and *I*_off_ are 590 nA and 0.1 pA, respectively, Figure 2b,c displays the simulation results corresponding to XNOR outputs of +1 and −1. When the XNOR output is +1, the CSA identifies the NAND cell’s *I*_on_, leading to a read access time of 2 ns. Conversely, for an XNOR output of −1, the CSA detects *I*_REF_, resulting in an prolonged read access time of 12 ns.

To mitigate the BER, Lee et al. proposed a 4T2S cell string architecture, which consists of two NAND cell strings and four input transistors, to facilitate XNOR operations in BNNs. This 4T2S architecture incorporates a differential sensing scheme within the cell string. Each synaptic string is composed of two NAND strings with synaptic cells connected in series, and it includes four input transistors that receive *V*_in1_ and *V*_in2_ at their respective gates. A key advantage of this design is that a single sense amplifier is paired with the four input transistors, resulting in a more streamlined structure compared with the synaptic configuration depicted in Figure 8.

**Figure 10:**
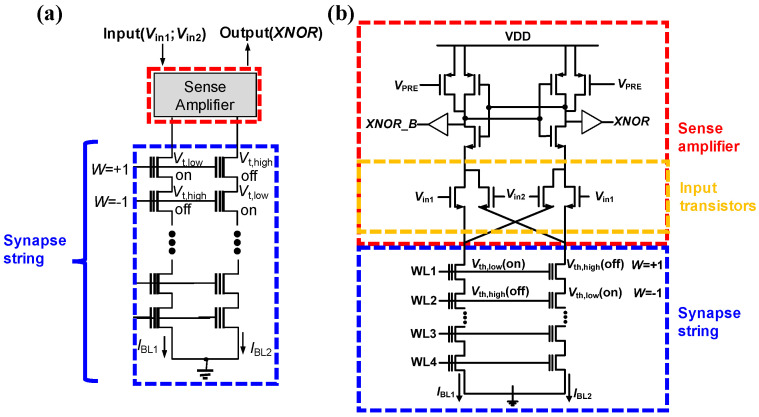
Differential sensing scheme based on the 4T2S synaptic string structure, which includes two NAND strings and four input transistors. Reprinted/adapted with permission from Ref. [67]. Copyright © 2022, Elsevier.

Figure 10a illustrates a synaptic string linked to a sense amplifier, while Figure 10b shows the differential current sense amplifier (DCSA). The DCSA is composed of two pre-charge PMOSFETs, a cross-coupled inverter pair, and four input transistors, with *V*_in1_ and *V*_in2_ imposed on the gates of the input transistors. A key feature of this design is that every synapses within a single synaptic string share the four input transistors, thereby decreasing the overall count of input transistors relative to the previous design in [82]. The DCSA operates by comparing the bit-line currents (*I*_BL1_ and *I*_BL2_) of two NAND flash cells to produce an XNOR output. This differential sensing method removes the necessity for fixed reference current circuits, which were demanded in the design expressed in Figure 8. Additionally, the DCSA extends its capability to perform logic operations, thereby minimizing CMOS overhead. It simultaneously functions as a reader for NAND flash cell currents and an XNOR operation processor. Each synapse, consisting of two adjacent NAND cells, adopts the approach presented in Figure 8 to encode synaptic weights. For input representation, a value of +1 is represented by applying complementary input voltages, where *V*_in1_ and *V*_in2_ correspond to *V*_on_ and *V*_off_, respectively. In contrast, an input of −1 is represented by reversing these input voltage patterns.

**Figure 11:**
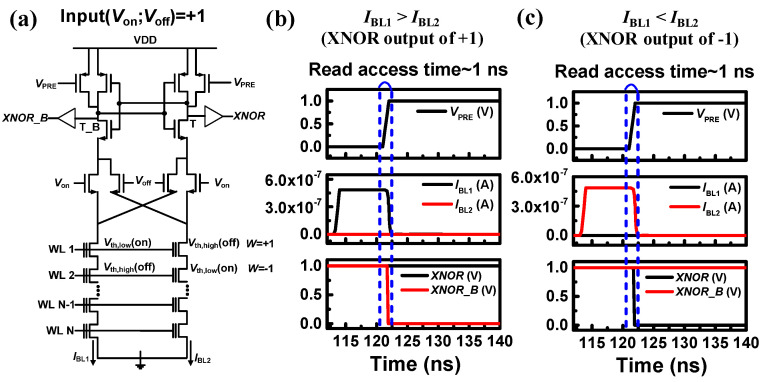
(**a**) DCSA with 4T2S structure when the input is +1. The transient waveforms (**b**) for the case where *I*_BL1_ is larger than *I*_BL2_ and (**c**) for the case where *I*_BL1_ is smaller than *I*_BL2_. Reprinted/adapted with permission from Ref. [67]. Copyright © 2022, Elsevier.

Figure 11a illustrates the DCSA linked to a synapse string when the input is +1 (*V*_in1_ = *V*_on_, *V*_in2_ = *V*_off_). Circuit simulations were performed utilizing a 20 nm FinFET-based BSIM-CMG model [83], and the bit-line currents (*I*_BL_) of the NAND flash cells. Figure 11b and 11c display the transient waveforms corresponding to XNOR outputs of +1 and −1, respectively. According to the measurement results, the measured *I*_on_ and *I*_off_ values of the NAND cells are 480 nA and 1.4 pA, respectively.

When the synaptic weight is +1, *I*_BL1_ is greater than *I*_BL2_ (*I*_BL1_ > *I*_BL2_), prompting the DCSA to generate an XNOR output of +1 with a read access time of approximately 1 ns. On the other hand, when the synaptic weight is −1, *I*_BL2_ exceeds *I*_BL1_ (*I*_BL2_ > *I*_BL1_), leading to the DCSA producing an XNOR output of −1, with a comparable read access time of around 1 ns. Notably, the DCSA demonstrates faster sensing speeds compared to the sense amplifier utilizing a fixed reference current that is shown in Figure 9. This is because the DCSA detects the *I*_on_ when the XNOR output is −1, whereas the CSA in Figure 9 detects the *I*_REF_, which slows down the sensing process.

**Figure 12:**
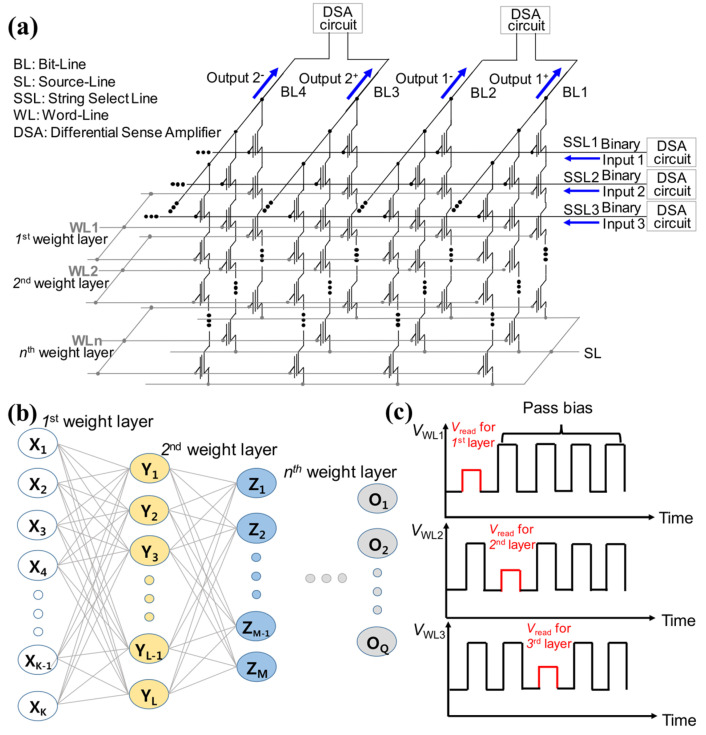
(**a**) Operation method of cell string structure to be used in QNN. (**b**) Schematic representation of a neural network. (**c**) Timing diagram illustrating the read pulse scheme as a function of time. Reprinted/adapted with permission from Ref. [68]. Copyright © 2020, IEEE.

Multi-bit quantized neural networks (QNNs) offer higher classification accuracy compared to BNNs and achieve performances similar to neural networks with floating-point weights. Lee et al. proposed NAND flash-based novel synaptic architecture for highly robust and high-density quantized neural networks with binary neuron activation of (1, 0) [68]. Figure 12a presents the proposed synaptic architecture for quantized neural networks (QNNs), which utilizes NAND flash memory combined with a differential sense amplifier (DSA). The NAND flash memory can use either a charge-trap layer or a floating-gate to preserve charge, and the proposed structure is compatible with both. However, this work specifically focuses on applying the architecture to 3D NAND flash memory having a charge-trap layer. Input voltages generated by the DSA circuits are imposed on the string-select lines (SSLs), while the resulting string current is aggregated along BLs, as illustrated in Figure 12a.

**Figure 13:**
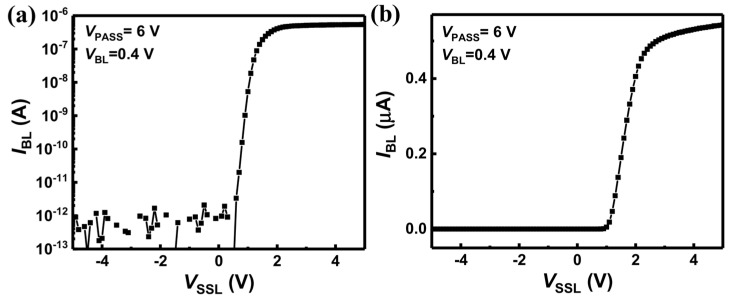
*I*_BL_-*V*_SSL_ characteristics shown in (**a**) logarithmic scale and (**b**) linear scale. Reprinted/adapted with permission from Ref. [68]. Copyright © 2020, IEEE.

In neuromorphic systems, vector–matrix multiplication is performed through a memory devices by assigning weights and input values in a deep neural network (DNN) to the resistance of the neuromorphic electronics and input voltage, respectively. Because the input voltage is imposed on the SSL, the I–V characteristics of the device must be taken into account. Figure 13a,b show the measured *I*_BL_ *V*_SSL_ in logarithmic and linear scales, respectively, indicating a nonlinear relationship between *I*_BL_ and *V*_SSL_.

However, in the DNN model, the multiplication and accumulation result exhibits a linear relationship with the input value. This discrepancy prevents encoding the input amplitude in the DNN model as an analog input voltage in neuromorphic systems. To address this, binary activation (1, 0) is adopted, where an input of 1 corresponds to the turn-on voltage (*V*_on_) and an input of 0 corresponds to the turn-off voltage (*V*_off_) of the SSL device. Binary input is advantageous because it decreases the complexity of neuron circuits and has been shown to achieve satisfactory accuracy in QNNs across various recognition tasks [84,85]. Figure 12b,c display the schematic of quantized neural networks and the read pulse scheme over time, respectively.

In this architecture, the NAND cells linked to the *n*th WL in Figure 12a act as synapses in the *n*th synapse layer shown in Figure 12b. The read bias voltage (*V*_read_) is applied sequentially to the selected word-lines, while a pass bias voltage (*V*_PASS_) is applied to the unselected word-lines, as illustrated in Figure 12c. The charge preserved in the cells linked to the selected WL, where *V*_read_ is imposed, decide the resulting current.

Unlike traditional NAND flash memory, where the *V*_on_ is imposed sequentially on each SSL to access memory conductance, this proposed scheme applies input voltages simultaneously to all SSLs. This parallel approach decreases read time relative to traditional NAND flash operations. Consequently, the output for the post-synaptic neuron layer is produced each time the *V*_read_ is imposed sequentially on the word-lines along the NAND string.

**Figure 14:**
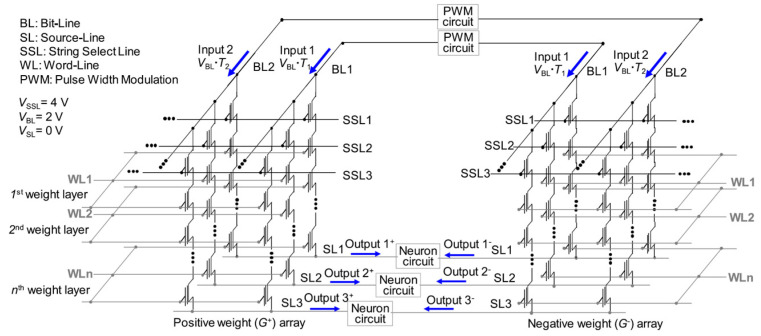
Synaptic array architecture utilizing 3D cell string structure for forward propagation (FP) process. Reprinted/adapted with permission from Ref. [69]. Copyright © 2021, IEEE.

**Figure 15:**
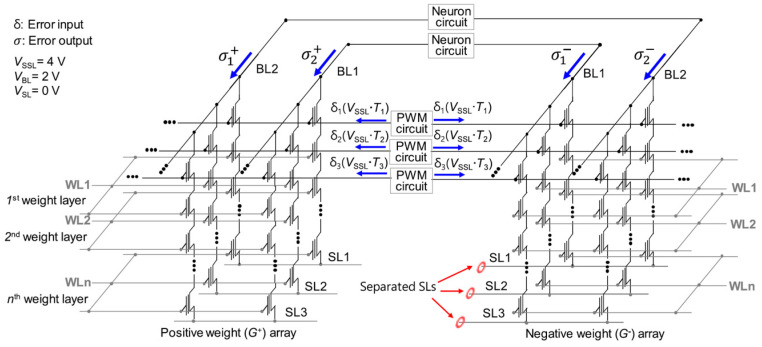
Synaptic array architecture utilizing 3D cell string structure for backward propagation (BP) process. Reprinted/adapted with permission from Ref. [69]. Copyright © 2021, IEEE.

On-chip learning presents a significant advantage by reducing both training energy and time consumption compared to off-chip learning. Furthermore, it allows for the compensation of weight variations in neuromorphic electronics and enhances adaptability to dynamic changing environments [86]. To facilitate on-chip learning, Lee et al. introduced an approach to utilize cell string structure performing on-chip learning [69]. Figure 14 and Figure 15 illustrate the operations for NAND flash memory to perform on-chip learning.

In traditional NAND flash memory, source lines (SLs) are connected within a block, which prevents efficient on-chip learning. To address this limitation, the proposed synaptic architecture separates SLs perpendicularly to the bit-lines (BLs), enabling FP and BP to occur in the same synaptic array. Two NAND cells, located in different synaptic weight arrays (*G*^+^ array and *G*^−^ array), are harnessed together to encode a negative weight.

During FP, input biases are imposed on the BLs, and the resulting weighted sum current flows through the separated SLs. In BP, error inputs (δ) are imposed on the string-select lines (SSLs), and the corresponding weighted sum current (s) flows through the BLs. In conventional approaches, such as in RRAM arrays [87], error inputs are imposed on SLs while currents are read from BLs. However, this approach can cause variations in the cell current between FP and BP due to the resistance of pass cells, which depends on the cell’s position in the string.

To ensure consistent string currents in both FP and BP, error inputs are imposed on SSLs, and the *V*_SL_, *V*_SSL_, and *V*_BL_ must maintain the same amplitudes during both propagation stages. This consistency is achieved by utilizing pulse-width modulation (PWM) circuits to produce width-modulated pulses having a fixed amplitude [88]. By imposing these pulses on BL during FP and SSL during BP, precise vector–matrix multiplication (VMM) is performed, effectively mitigating the effect of pass cell resistance in the 3D cell string structure.

Worth noting, *I*_BL_ flows in the same direction during both FP and BP. In this scheme, *V*_SSL_ is imposed ono all SSLs to gather currents from memory cells through the BLs. This approach contrasts with traditional NAND flash memory operation, where *V*_SSL_ is imposed only on a selected SSL to access data in a single NAND cell. As a result, the proposed method improves read speed relative to traditional NAND flash memory operations.

## 5. Discussion and Conclusions

This review has explored recent advancements in neuromorphic computing using flash memory, focusing on NOR, AND, and NAND flash memory technologies. Each type of flash memory exhibits unique strengths and limitations, making them suitable for different applications within neuromorphic systems.

In NOR flash memory, each memory cell is connected directly to the BLs through individual contacts, resulting in lower cell density compared to AND and NAND flash memory. Additionally, NOR flash memory requires individual source and drain contacts for each memory cell, making it challenging to implement three-dimensional (3D) vertical structures. Consequently, its integration density is lower than that of AND and NAND flash memory, which are capable of 3D memory implementation. However, NOR flash memory has the advantage of enabling both forward propagation and backward propagation within the same array, because the BLs run perpendicular to the SLs in NOR flash memory. Specifically, forward propagation can be performed by imposing input voltages on the source lines, while backward propagation can be performed by imposing error input voltages on the bit lines, allowing both operations to take place within the same array.

Next, AND flash memory supports 3D integration but requires horizontally oriented source and bit lines. This structural limitation makes it difficult to perform forward and backward propagation within the same array, posing challenges for on-chip learning. While recent studies have proposed using direct feedback alignment to enable on-chip learning with AND flash memory, this approach necessitates the use of separate synaptic arrays for forward and backward propagation. As a result, the overall area occupied by the synaptic devices increases, which is a notable drawback.

Finally, NAND flash memory supports 3D integration and achieves the highest integration density among the three types due to its serial connection of cells between the source and bit line. Its structural design allows for highly compact 3D memory implementation, making it a preferred choice for dense memory arrays. The synaptic architecture for executing XNOR operations in binary neural networks (BNNs) have been introduced. While this structure effectively performs XNOR operations in BNNs, when the XNOR output is −1, it experiences a prolonged sensing time of 12 ns. This delay results from the sensing scheme’s dependence on a reference current source. To overcome these limitations and enhance read speed while reducing BER, Lee et al. proposed the 4T2S architecture, which harnesses a differential sensing scheme. Compared to the fixed reference current scheme, the differential sensing approach provides a faster sensing speed when the XNOR output is −1, as it directly detects *I*_on_ instead of *I*_REF_. Furthermore, the differential sensing scheme improves accuracy by lowering the bit-error rate thanks to its inherent differential architecture. Compared to the 2T2S structure, The 4T2S design also streamlines the synaptic string architecture and removes the necessity of a reference current source. Lee et al. introduced an innovative approach that enables both forward propagation (FP) and backward propagation (BP) within the same 3D cell string structure. This is achieved by separating the SLs perpendicular to the BLs. However, this configuration increases voltage drop and RC delay, resulting in higher read time and reduced accuracy in vector-matrix multiplication computations. To mitigate these effects, on-chip learning should employ the conventional NAND flash memory structure, where SLs remain unified rather than separated. In response, Lee et al. proposed utilizing the cell stringstructure to support error backpropagation in synaptic architectures with random synaptic feedback weights [89]. To enable error backpropagation, forward and backward propagations are processed in separate synaptic devices in forward and backward synaptic arrays, respectively. In addition, synaptic weights in a forward synaptic array are updated at each iteration, while those in a backward synaptic array are fixed to reduce burden of peripheral circuits and power consumption.

The overall analysis of the flash memory based neuromorphic system is shownin Table 1.

Flash memory-based neuromorphic computing continues to be a promising area of research, offering scalable and efficient solutions for overcoming the limitations of traditional computing architectures. Future studies should focus on addressing the challenges associated with each type of flash memory to unlock their full potential in neuromorphic applications.

## Figures and Tables

**Table 1:** Analysis of flash memory based neuromorphic systems.

Reference	Learning Type	Input Terminal	Output Terminal	Operation Voltage	Pass Bias	On-Current (*I*_BL_)	The Number of WL Floors	Input, Weight Resolution	Characteristics	Memory Architecture
[54]	Off-chip Learning	Gate of NOR flash memory	Source of NOR flash memory	2.7 V	-	~100 nA	Up to 785	Multi-bit weight, binary input	1. Utilizing the energy-saving gate coupling of the peripheral and array cells 2. Having a nearly exponential dependence of the output current of the memory cell on the input voltage	NOR
[55]	On-chip Learning	Gate of NOR flash memory	Drain of NOR flash memory	1 V	-	~10 nA	1	Multi-bit weight, binary input	1. Propose device using a thin-film transistor (TFT)-type NOR flash memory cell with a half-covered floating gate. 2. The structure can implement the STDP characteristic of a synapse without any additional circuit configuration.	NOR
[61]	Off-chip learning	Gate of AND flash memory	Bit-line	1 V	-	~10 nA	3	Binary weight, binary input	1. Low-power operation is possible with a semicircular poly-Si channel surrounded by a single word-line. 2. In each floor, two cells facing each other along the long axis of a single channel hole share a source/drain, greatly improving cell density and making it suitable for high-density BNNs.	AND
[67,70]	Off-chip learning	Gate of input transistor	Bit-line	0.4 V	6 V	600 nA	64	Binary input, Binary weight,	1. [70]: Develop a method to utilize 2T2S in BNN 2. [67]: Develop a method to utilize 4T2S in BNN 3. 4T2S decreases latency and BER relative to the 2T2S.	NAND
[68]	Off-chip learning	String-select line	Bit-line	2 V	6 V	<1.4 uA	64	Binary input, Multi-bit weight	1. Utilizing binary neuron activation to reduce burden of circuits 2. Reading in saturation region of NAND cells can eliminate effect of pass cells.	NAND
[69]	On-chip learning	Forward: Bit-line Backward: String-select line	Forward: Source-line Backward: Bit-line	2 V	6 V	200 nA	64	Multi-bit input, Multi-bit weight	1. Develop a method to utilize NAND flash memory in on-chip 2. Using a single program pulse in each weight update	NAND

## Data Availability

Not applicable.
